# CAREGIVER STATUS MODERATION OF THE RELATION BETWEEN PERCEPTIONS OF COGNITION AND COGNITIVE PERFORMANCE

**DOI:** 10.1093/geroni/igae098.3268

**Published:** 2024-12-31

**Authors:** Maia McLin, Danielle Nadorff

**Affiliations:** Mississippi State University, Mississippi State, Mississippi, United States; Mississippi State University, Mississippi State, Mississippi, United States

## Abstract

Custodial grandparents have additional stressors associated with becoming a caregiver later in life. Caregiving grandparents use cognitive strategies that non-caregiving older adults may not regularly utilize. Concerns regarding aging are common to older adults, however these concerns may be elevated amongst older adults who have the additional concern of responsibility over grandchildren. In general, those who report more control over cognitive aging are more likely to have better cognitive performance. These perceptions may affect behaviors such as participating in cognitively-taxing tasks. Those who have additional stressors, such as caregiving to grandchildren, may have additional burden that lessens perceptions of cognitive control. This study assesses whether caregiving grandparent status affects the relation between perceptions of control over aging and cognitive performance. Data included 234 caregiving and 1591 non-caregiving participants from the third wave of a longitudinal data collection survey Midlife in the United States study (MIDUS III). MIDUS III included self-administered surveys assessing caregiver status, social factors, and phone interview assessment of cognitive performance. Moderation of caregiver status on the relation between perceptions of cognition control and cognitive performance was tested using SPSS Process macro. A significant interaction was found (t=2.08, p<.05), indicating that caregiver status moderates this relation. Perceptions of cognitive control were not predictive of cognitive performance for grandparent caregivers (p=.06), but were predictive for non-caregivers (p<.001). These results suggest that grandparent caregiver status affects the impact of perceptions of cognitive control on cognitive performance.

